# Blood Immune Cells as Biomarkers in Long-Term Surviving Patients with Advanced Non-Small-Cell Lung Cancer Undergoing a Combined Immune/Chemotherapy

**DOI:** 10.3390/cancers15194873

**Published:** 2023-10-06

**Authors:** Miriam Möller, Wolfgang Schütte, Steffi Turzer, Barbara Seliger, Dagmar Riemann

**Affiliations:** 1 Clinic of Internal Medicine, Hospital Martha-Maria Halle-Dölau, 06120 Halle, Germany; miriam.moeller@martha-maria.de (M.M.); wolfgang.schuette@martha-maria.de (W.S.); 2 Institute of Medical Immunology, Martin Luther University Halle-Wittenberg, 06112 Halle, Germany; steffi.turzer@uk-halle.de (S.T.); barbara.seliger@uk-halle.de (B.S.); 3 Institute of Translational Immunology, Medical School “Theodor Fontane”, 14770 Brandenburg, Germany

**Keywords:** biomarkers, immune checkpoint blockade, non-small-cell lung cancer, long-term survival, neutrophil/lymphocyte ratio, HLA-DR^low^ monocytes, slan+ non-classical monocytes, dendritic cells

## Abstract

**Simple Summary:**

Tumor cells can evade recognition and killing via immune cells by expressing co-inhibitory membrane molecules, which suppress the activity of tumor-specific T cells. Immune checkpoint inhibitor (ICI) therapies act by blocking these inhibitory pathways via monoclonal antibodies. Due to the limited efficacy of ICI therapy, biomarkers have to be detected, a process that can identify patients who benefit from this long-term treatment. In this pilot study, immune monitoring of four blood cell markers was performed over time in advanced non-small cell lung cancer (NSCLC) patients undergoing combined immune/chemotherapy and surviving ≥12 months. We demonstrate that a low neutrophil/lymphocyte ratio (NLR), a low percentage of suppressive HLA-DR^low^ monocytes, and/or clearly detectable numbers of slan+ non-classical monocytes and of dendritic cells can be predictive markers for therapy responses and treatment outcomes. These markers might have an impact on the treatment decisions for NSCLC patients, but need to be validated in larger cohorts.

**Abstract:**

An important challenge remains in identifying the baseline characteristics of cancer patients who will mostly benefit from immune checkpoint inhibitor (ICI) therapies. Furthermore, biomarkers could help in the choice of an optimal therapy duration after a primary therapy response. In this pilot study, the time courses of four different immune cell parameters were followed in 12 patients with advanced non-small-cell lung cancer (NSCLC) undergoing ICI therapy combined with chemotherapy and surviving at least 12 months. Blood was collected at the time point of the first and third antibody administration, as well as after 12 months of patients’ survival. Using multi-color flow cytometry, two suppressive markers (neutrophil/lymphocyte ratio (NLR) and the frequency of circulating HLA-DR^low^ monocytes), as well as two markers of an ongoing immune response (6-Sulfo LacNAc (slan)+ non-classical monocytes and dendritic cell (DC) subtypes), were determined. In most of those who survived > 12 months, a low NLR and a low number of HLA-DR^low^ monocytes combined with clearly detectable numbers of slan+ non-classical monocytes and of DC subtypes were seen. Two of the patients had an increase in the suppressive markers paired with a decrease in slan+ non-classical monocytes and in DC subtypes, which, in at least one patient, was the correlate of an ongoing clinical progression. Our results implicate that the NLR, specific subtypes of monocytes, and the number of blood DCs might be useful predictive biomarkers for cancer patients during long-term treatment with ICI/chemotherapy.

## 1. Introduction

Despite modest responses to platinum-based chemotherapy and short intervals until disease progression [1,2], these agents were used as standard forms of therapy for patients with advanced non-small-cell lung cancer (NSCLC) for many years. In the past decade, immune checkpoint inhibitors (ICIs) targeting the PD-L1/PD-1 signaling axis have emerged as a novel treatment option for lung cancer patients, though only a limited proportion of patients can benefit from them [3,4]. The therapeutic activity of ICIs is the result of a complex interplay between multiple cells and soluble factors in the tumor microenvironment (TME) and the immune system (for review, see [5]). Based on the ICI which was employed, two distinct activities have been reported: (i) competition for the ligands of co-inhibitory receptors and (ii) regulation of the surface expression of these receptors. ICIs’ efficacy depends on the tumor mutational burden [6], the cellular heterogeneity associated with patterns of immune suppression [7], and the tumor cell expression of immune checkpoint molecules (for review see [8]). With respect to patients’ prognosis, the immunotherapy response patterns, immune-related adverse events, and tumor stages are associated with the diversity of the continuation of ICI therapy in previous studies, but there exists no clear consensus on the optimal duration of ICI therapy [9]. Biomarkers of ongoing immune response/disease progression could help to guide the long-term therapeutic regimen and could optimize ICI-based combination therapies. Blood biomarkers have difficulty in reflecting the TME, including the spatial distribution of tumor and immune cells, but are easier to handle than tumor tissues/biopsies. Blood-based cellular immune biomarkers have shown great promise in predicting responses to ICI therapies due to easy specimen accessibility, the opportunity for serial monitoring, quantitative measurement, and the availability of unique analytic high-throughput (single cell) platforms [10]. In a recent study [11], we found that NSCLC patients with a baseline neutrophil-to-lymphocyte ratio (NLR) ≥ 6.1, ≥ 22% HLA-DR^low^ monocytes, a frequency of slan+ non-classical monocytes < 0.25% of leukocytes, and/or amounts of dendritic cells (DC) ≤ 0.14% of leukocytes showed poor progression-free survival (PFS). In this study, the four blood cell biomarkers were monitored in patients with advanced NSCLC surviving at least 12 months of a combined ICI/chemotherapy to investigate whether these markers are useful to predict the treatment outcomes of patients.

## 2. Materials and Methods

*Patients’ cohort.* The study was approved by the institutional review board of the Aerztekammer Sachsen-Anhalt (69/18). All patients gave written informed consent for the study’s proposal and procedures. In a former study, 90 patients with histologically confirmed unresectable locally advanced or metastatic lung cancer, prior to PD-1 or PD-L1 blockade treatment in combination with chemotherapy, were prospectively enrolled from June 2019 to June 2021 (Table 1 in [11]). In total, 12 of the 36 patients surviving ≥ 12 months consented to a third blood draw and were included in the current pilot study. All patients were >18 years old and had a histologically confirmed diagnosis of advanced lung cancer, adequate organ function, and the capacity to make an informed decision. Patients with an active autoimmune disease were excluded from the study, as were patients with treatable oncogenic driver alterations. A combined immune/chemotherapy with nab-paclitaxel, carboplatin, and pembrolizumab was given to patients with squamous cell carcinoma (SqC), according to the KEYNOTE-407 trial [12], but also to patients with thyroid transcription factor (TTF)-1-negative adenocarcinoma (AC), as pemetrexed might have been less effective in these patients [13]. Patients with AC and liver metastases received a combined immune/chemotherapy with atezolizumab, bevacizumab, carboplatin, and nab-paclitaxel, since this combination had a clear advantage in terms of the presence of liver metastases, according to the IMpower150 study [14]. All other AC patients received a combined immune/chemotherapy with pemetrexed, carboplatin, and pembrolizumab (KEYNOTE-189 trial [15]). Therapy response rates were determined according to RECIST 1.1 criteria. PFS was defined as the time from the initiation of immune/chemotherapy to the first observation of cancer progression or death from any cause, whereas overall survival (OS) was defined as the time from the initiation of therapy until death from any cause.

*Blood samples, antibody staining, and flow cytometry.* Blood samples of a former study [11] were collected before the initiation of immune/chemotherapy (time point 0, baseline) and prior to the third therapy cycle (t_1_). For the current pilot study, 12 patients with an OS of at least 12 months consented to a third blood draw (t_2_). Leukocyte count and complete blood count were measured with a routine hematology analyzer. Circulating DC subpopulations were determined with the “Blood DC Enumeration Kit” (Miltenyi, Bergisch Gladbach, Germany), which was supplemented for gating reasons with CD45-APC-H7 and a HLA-DR-V500 monoclonal antibody (mAb). Briefly, 300 µL of blood was incubated with mAb CD141/BDCA-3-APC as a marker for myeloid conventional DC (cDC1), anti-CD1 c-PE (cDC2), and CD303/BDCA-2-FITC for plasmacytoid DC (pDC) [16]. At least 1 × 10^6^ leukocytes were analyzed on a FACS CANTO II Flow Cytometer (BD Biosciences, Heidelberg, Germany) using a recently published gating strategy [11]. Monocytic HLA-DR intensity was quantified using a mAb labeled on a protein/fluorophore ratio of 1/1 (clone L243; QuantiBRITE™ reagent; BD Biosciences), according to the manufacturer’s instructions. A standard curve for antigen quantification was established with QuantiBRITE beads (BD Biosciences) to convert the measured geometric mean fluorescence intensity (MFI) of the gated monocytes into “antibody molecules bound per cell” (ABC) values. Monocytic HLA-DR MFI values of ≤5000 ABC were designated as “immunoparalysis”, since the patients were at high risk of infectious diseases [17]. The percentage of HLA-DR^low^ monocytes was estimated by taking a MFI of 5000 ABC as the cut-off value for a low HLA-DR intensity [18].

For the labeling of monocytes/lymphocytes, 300 µL blood was stained with mAbs specific to slan (M-DC8)-FITC (Miltenyi Biotec, Bergisch Gladbach, Germany); CD16-PE-Cy7 (Biolegend, San Diego, CA, USA); and CD19-PerCP-Cy5.5 (InVitrogen/Thermo Fisher, Waltham, MA, USA). All other mAbs (CD14-APC, CD45-APC-H7, CD3-V450, HLA-DR-V500) were obtained from BD Biosciences (Heidelberg, Germany). After two washing steps, the cells were analyzed with the FACS CANTO II (BD Biosciences, Heidelberg, Germany). The gating strategy for slan+ non-classical monocytes is provided in [11]. Data analysis was performed using the BD FACSDiva^TM^ software V8.0.1. Cytometer Setup and Tracking (CST) Beads (BD Biosciences) were used daily to set standardized geometric MFI ranges in the fluorescence channels which were used.

## 3. Results

### 3.1. Patient Characteristics

The general baseline characteristics of the 12 NSCLC patients surviving at least 12 months and who were available for a third blood draw are summarized in Table 1. Concerning the cohort of patients, 10/12 patients had AC histology, and 11/12 patients were male. Patients were between 48 and 84 years old. PD-L1 tumor expression ranged between zero and 100%, and complete remission was associated both with 0% and with 100% PD-L1 expression. Patients received 15–34 cycles of ICI therapy. Of the 12 patients, 5 patients were still living at the time of the evaluation of this study in January 2023, with complete remission in four patients. The other patients survived 5–18 months after the third blood draw (shown as OS3 in Table 1).

### 3.2. The Time Course of Blood Cell Markers

Blood samples from the 12 NSCLC patients were monitored over time. Table 2 summarizes the four blood cell markers measured at baseline (t_0_), both at the third ICI application (t_1_) and after surviving at least one year (t_2_). The baseline values associated with poor PFS were described in a recent study [11] to be a NLR ≥ 6.1, HLA-DR^low^ myeloid-derived suppressor cells (MDSC) ≥ 22% of monocytes, slan+ non-classical monocytes < 0.25% of leukocytes, and/or DC levels ≤ 0.14% of leukocytes. With two exceptions (patients #6 and #11), most of the 12 patients had a baseline NLR below the critical value of 6.1 (mean 4.3). At the time point of the third ICI application, the NLR values of all patients dropped below 6.1. This result can be interpreted as an initial therapy response, since patients with tumor progress showed an NLR increase at t_1_ in the former study [11]. Similar results were obtained for HLA-DR^low^ MDSC: Only 2/12 patients (patients #3 and #11) exhibited baseline levels ≥ 22% of monocytes, which had declined by the time of the third ICI cycle. Interestingly, the high baseline levels of HLA-DR^low^ monocytes were associated with low percentages of slan+ monocytes (<0.25% of leukocytes and <2% of monocytes), and additionally, in patient #11, with low DC levels. Patient #11 had an OS of 15 months despite poor baseline markers and the presence of brain/liver metastases (Table 1). Our results show that although high baseline values of immunosuppressive neutrophils and monocytes are a risk factor for a lacking therapy response and a poor PFS [11], patients can survive > 12 months if they have ameliorated their blood cell markers by the third ICI cycle.

**Figure 1:**
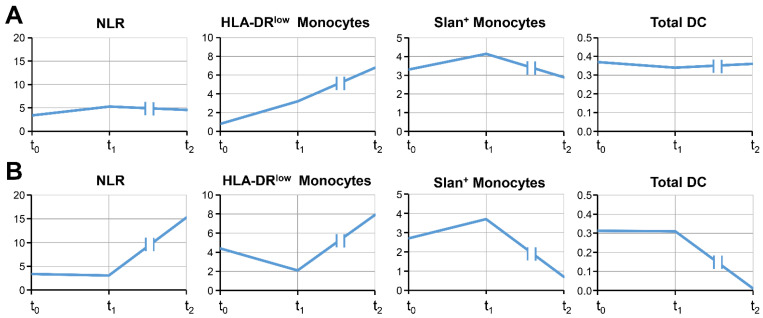
Time course of the four biomarkers NLR, HLA-DR^low^ monocytes, slan+ non-classical monocytes, and total DC in one patient with complete remission (**A**) and in one patient with disease progression (**B**). The time points given at the X-axis are the baseline values (t_0_), the time of the third ICI application (t_1_), and a time point after at least 12 months OS (t_2_). HLA-DR^low^ and slan+ non-classical monocytes are given as % of monocytes; the number of DC is given as % of leukocytes.

In total, 4 of the 12 patients with a third blood collection went into complete remission. After at least 12 months of the patients’ survival (t_2_), these patients showed low blood cell counts associated with immune suppression (NLR < 6.1 and/or <10% of monocytes with a HLA-DR^low^ phenotype) and clearly detectable markers of an ongoing immune response (2.8–11.8% slan+ non-classical monocytes and/or DC levels of ≥0.16% of leukocytes) (Table 2). The marker values of the third cycle (t_1_) and after 12 months of patients’ survival (t_2_) were stable and comparable in most cases, as displayed by arrows in Table 2. Figure 1A illustrates the time course of the four blood markers in patient #1 as an example of a long-term survivor with complete remission, demonstrating relatively stable blood cell values.

Another dynamic pattern was found in patient #4 (Figure 1B), who underwent the third blood draw at a time at which progressive disease had been proven. This patient had a lymphopenia (590 lymphocytes/µL blood), resulting in a high NLR of 15.2. Furthermore, the numbers of DC were close to zero, and the slan+ non-classical monocytes decreased below baseline levels, while HLA-DR^low^ MDSC increased, but remained <10% of monocytes. This patient died 5 months after the third blood collection (OS 25 months). A similar dynamic could be observed in patient #3, who had relatively high levels of HLA-DR^low^ MDSC over time (Table 2). At t_2_, an increase in both the NLR and in HLA-DR^low^ MDSC above baseline levels was noticed. Although slan+ non-classical monocytes showed stable values, DC counts decreased. Nine months after the third blood collection, tumor progression was recognized in this patient, who died 3 months later (OS 34 months). The examples of patients #3 and #4 show that a deterioration of blood cell markers could indicate worsening of the tumor disease.

Interestingly, patient #12 (complete remission) had a high number of slan+ non-classical monocytes over the time course of all blood collections (Table 2). The patient’s slan+ non-classical monocytes, known to exhibit anti-tumor activity, surpassed the number of HLA-DR^low^ MDSC in the blood. In three of the five patients still living at the end of our study, the quotient of slan+ non-classical monocytes/HLA-DR^low^ monocytes had values > 1 (Table 3). However, for most of the patients of this study, the monocytic quotient showed values < 1, suggesting a preponderance of monocytic MDSC in blood, even in patients with complete remission, such as in patient #1 and #2. Based on our data, it could be assumed that there exists an individual equilibrium of the different monocytic subpopulations in the blood. Tumor progression was associated with a very low quotient of slan+ non-classical monocytes/HLA-DR^low^ monocytes, as shown in patients #3 and #4. The ratio of slan+ non-classical monocytes/HLA-DR^low^ monocytes could be a hopeful new marker for the monitoring of tumor patients.

**Figure 2:**
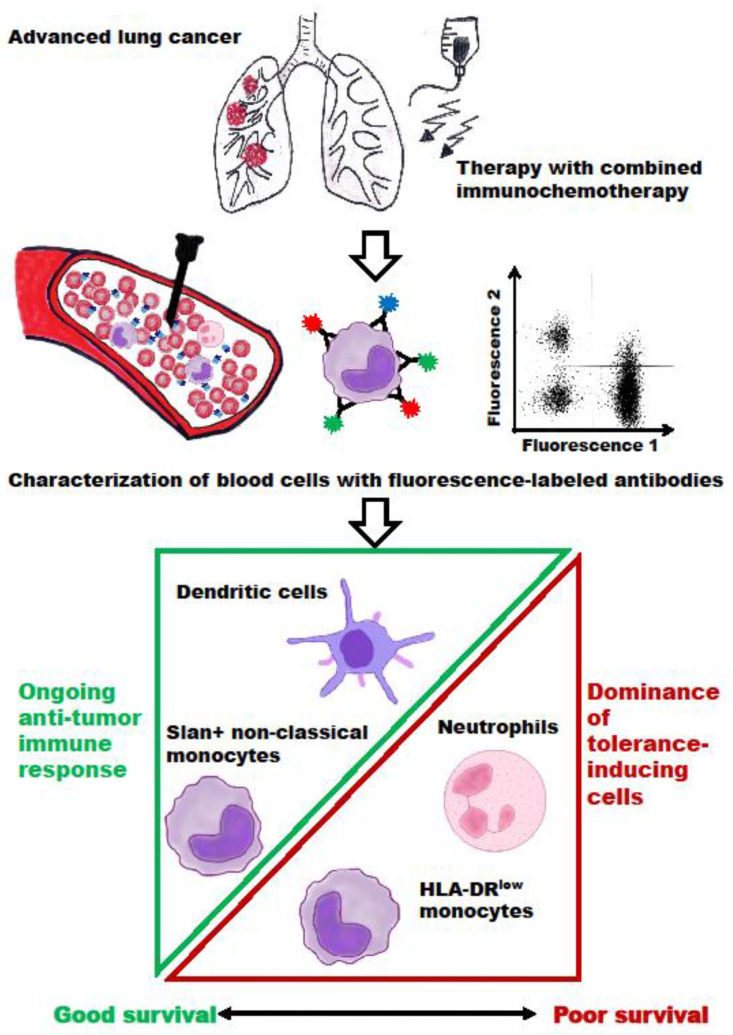
Schematic diagram of the immune monitoring process of this study. In patients with advanced NSCLC undergoing immune/chemotherapy, blood cells were stained using fluorescence-labeled mAb. For the monitoring of therapy-induced changes in immunogenicity, DC and slan+ non-classical monocytes, as well as neutrophil/lymphocyte ratio and HLA-DR^low^ monocytes, were quantified. A good patient survival rate was associated with an ongoing anti-tumor response with detectable amounts of DC and slan+ non-classical monocytes. The dominance of tolerance-inducing neutrophils and HLA-DR^low^ monocytes reflects the resistance to immune/chemotherapy and poor survival.

Taken together, our results implicate that the NLR, specific subtypes of monocytes, and the DC counts might be useful biomarkers for the long-time monitoring of NSCLC patients’ survival. As illustrated in Figure 2, an ongoing and effective anti-tumor response is associated with both a detectable proportion of slan+ non-classical monocytes and of DC, resulting in better patient survival. In contrast, a high NLR (neutrophils dominate over lymphocytes) and a high percentage of HLA-DR^low^ MDSC are signs of immune tolerance, tumor progression, and poor patient survival.

## 4. Discussion

The implementation of ICI therapy has revolutionized lung cancer therapy, with significant survival benefits for patients with advanced wild-type NSCLC. However, since not all patients with advanced and/or metastatic disease can benefit from ICI therapy, predictive biomarkers are urgently needed [19]. In ICI monotherapy, a good ECOG score, PD-L1 tumor expression of ≥50%, an absence of bone metastasis, and the presence of skin toxicity have been correlated with a good PFS and OS [20]. Since the response to ICI therapy is complex, single biomarkers are rarely able to precisely predict therapy responses. Thus, a combination of multiple biomarkers could be helpful, as demonstrated by the increased performance using a combination of PD-L1 immunohistochemistry, T-cell infiltration, and assessment of tumor mutational burden when compared with the three parameters alone [21].

Immune-related therapies can prolong the median OS of advanced wild-type NSCLC to 17–22 months, and a pooled OS of 16.2 months could be determined as the long-term survival standard (for review, see [22]). One of the major issues with ICI therapy is the determination of the optimal treatment duration [9]. Immune response characteristics are the basis of the unprecedented long-term survival of lung cancer patients with ICI/chemotherapy. Therefore, biomarkers of an ongoing immune response could help to guide long-term therapeutic regimens and could optimize ICI-based combination therapies. Our present pilot study confirms the usefulness of selected blood immune cells as biomarkers in NSCLC patients with advanced tumor stages and/or metastasis surviving at least 12 months. Comparable to the baseline marker expression, the increases in NLR and HLA-DR^low^ MDSC, as well as the decreases in tumor-fighting slan+ non-classical monocytes and DC, could be found during the time course of therapy, and were associated with a declining immune response and ongoing tumor progression. In contrast, all four patients with complete remission exhibited a NLR < 6, HLA-DR^low^ MDSC <10% of monocytes, slan+ non-classical monocytes of >2% of monocytes, and DC numbers ≥0.16% of leukocytes.

In the resistance to immunotherapy and the tumor-driven down-regulation of the immune response, several cell types are involved and can be monitored. The NLR combining neutrophils and lymphocytes is an established marker for the prognosis of lung cancer patients [8,23,24]. Neutrophils contain a subpopulation that promotes tumor growth and metastasis, stimulates angiogenesis, and mediates immunosuppression [25]. An enrichment in neutrophil-related proteins was observed in NSCLC patients with lacking responses to ICI therapy [26]. Similarly, HLA-DR^low^ monocytes are known to suppress lymphocytic functions in cancer patients [27,28,29]: in patients with hepatocellular carcinoma, HLA-DR^low^ MDSC inhibits NK cell cytotoxicity and cytokine secretion [30]. Several mediators and cytokines can reduce monocytic HLA-DR intensity, including IL-10, which increases the intracellular sequestration of MHC class II molecules via ubiquitination [31], or TGF-ß, which down-regulates the transcription of HLA-DR through the class II transactivator (CIITA) [32]. TGF-ß might be involved in the resistance to chemotherapy and/or ICI therapy [33], as a continuation of the TGF-β inhibitor with immunotherapy showed promising results in pre-clinical studies [34].

Monocytic subpopulations, such as classical (CD14^high^, CD16^−^), intermediate (CD14^high^, CD16^+^), and non-classical (CD14^low/−^, CD16^+^) monocytes, show transcriptomic differences that translate into specialization and different functions [35,36]. The CD16+ non-classical monocytes especially have been regarded as a pro-inflammatory population exhibiting tumor-killing properties [37]. Non-classical monocytes are crucial for ICI therapy of patients with malignant melanoma, since they are involved in the killing of regulatory T cells via the CTLA-4 mAb [38]. CD16^+^ non-classical monocytes can be further divided into slan+ and slan− populations [39,40]. Although they are of monocytic origin, slan+ cells may either rapidly acquire DC functions or differentiate into macrophages [41]. Slan+ monocytes can activate NK cells via IL-12, and the crosstalk between slan+ cells and NK cells improves the differentiation of naïve CD4^+^ T lymphocytes into T-helper type 1 (Th1) cells [42]. In patients with diffuse large B-cell lymphoma, slan+CD16+ non-classical monocytes, but not CD14^+^ monocytes, increased and displayed highly efficient, rituximab-mediated, antibody-dependent cellular cytotoxicity, almost equivalent to that exerted by NK cells [43]. 

DCs form a functional bridge between the innate and adaptive immune systems, and are important regulators of immune reactions. DCs are also important players in immunotherapy approaches. Strategies to harness the T-cell stimulatory function of DC for cancer immunotherapy aim at inducing antigen-specific T-cell responses of a Th1 phenotype accompanied by priming of cytotoxic T lymphocytes with the ability to eradicate tumor cells [44,45]. In the context of cancer, several alterations in the frequency and function of DCs have been reported [46,47,48]. The efficacy of ICI therapy was limited by a paucity of activated CD103+ DC in melanoma lesions [49], and a DC gene signature was strongly associated with improved patient OS in NSCLC patients undergoing atezolizumab therapy [50]. Blood DC can be divided into at least three distinct main subsets: conventional DC type I or II (cDC1 or cDC2, respectively) and pDC (for review see [51]). pDC is known for its ability to produce high amounts of type-I interferons upon stimulation via viral patterns, and has limited antigen-presenting potential. CD141^+^ cDC1 has superior antigen presentation activity on MHC class I molecules to CD8^+^ T cells, whereas CD1c^+^ cDC2 present antigens via MHC class II molecules to CD4^+^ T cells. A vaccine of cDC2 and pDC pulsed with tumor antigens was tested in metastatic melanoma and prostate cancer [52,53], leading to immunological responses and, in some patients, also to long-term survival. Furthermore, vaccination with naturally occurring cDC1s loaded with immunogenic cell-death-derived whole-tumor antigens could synergize with anti-PD1 treatment [44]. In a former study [11], we found that NSCLC patients undergoing combined immune/chemotherapy and possessing a DC baseline frequency of ≤0.14% of leukocytes had poor PFS. Age-matched healthy persons had DC levels of 0.32 ± 0.03% of leukocytes [47]. 

## 5. Conclusions 

Despite notable clinical responses, basic and clinical studies are still required in order to investigate the exact mechanism of ICI immunotherapy and to improve the appropriate selection of patients. In a pilot study, we demonstrated the value of a longitudinal monitoring of the immune surveillance in 12 NSCLC patients with an advanced tumor disease surviving at least 12 months while receiving immune/chemotherapy. Our results imply that the NLR, specific subtypes of monocytes, and blood DC counts might be useful biomarkers for the monitoring of NSCLC patients. An ongoing and effective anti-tumor response is associated with both a detectable proportion of slan+ non-classical monocytes and of DC subtypes, resulting in the better survival of patients. In contrast, dominating neutrophils and a high NLR, as well as a high percentage of HLA-DR^low^ MDSC, are signs of immune tolerance and tumor progression. The cellular blood biomarkers which we investigated might improve the prediction of the clinical and durable benefit of patients, and might provide insights into the underlying mechanisms of therapy resistance associated with reduced patient survival. It is mandatory that these blood cellular markers are further investigated and validated in larger multicenter patient cohorts as soon as possible.

## Figures and Tables

**Table 1:** Patients’ characteristics. Tumor histology, sex, age, metastases of brain/liver, PD-L1 status, ECOG performance status, smoker status, ICI cycle numbers until the end of the study, best therapy response (CR = complete remission; PR= partial remission; SD = stable disease; PD= progressive disease), overall survival (OS; in months), and survival time after third blood draw (OS3; in months) are shown. Patients still alive at the end of the study are highlighted in yellow; patients with complete remission in red.

	Histo	Sex	Age	Meta. Brain/Liver	PD-L1	ECOG	Smo	ICI Cycles	Response	OS	OS3
**1**	AC	M	67	0	0%	0	yes	33	CR	>38	>16
**2**	AC	M	62	yes	100%	0	yes	31	CR	>38	>16
**3**	AC	M	73	0	90%	1	yes	30	PR (PD 05/22)	34	12
**4**	AC	M	76	0	85%	1	yes	15	PR (PD 07/21)	25	5
**5**	AC	M	69	0	0%	1	yes	31	SD	29	11
**6**	SqC	M	84	0	0%	0	yes	29	CR	>35	>18
**7**	AC	M	71	0	10%	0	yes	27	SD	34	18
**8**	AC	F	56	0	0%	0	yes	34	PR	>32	>12
**9**	AC	M	79	0	0%	0	yes	17	PR	23	11
**10**	AC	M	48	0	0%	0	yes	19	PR	22	11
**11**	SqC	M	74	yes	70%	0	no	13	PR	15	5
**12**	AC	M	55	0	80%	1	yes	21	CR	>38	>18

**Table 2:** Cellular blood biomarkers in 12 NSCLC patients surviving at least 12 months over time. The biomarkers NLR, HLA-DR^low^ MDSC (as % of monocytes), slan+ non-classical monocytes (as % of monocytes), and sum of MDC/PDC (in % of leukocytes) were monitored over time. The time points given are the baseline values (t_0_), the third antibody application (t_1_), and after at least 12 months OS (t_2_). D shows the dynamics between t_1_ and t_2_. Values above/below the cut-off point for poor survival are marked in bold. Patients still alive at the end of the study are highlighted in yellow.

Pat. No.	Response	NLR				HLA-DR^low^ Monocytes				Slan+ Monocytes				MDC/PDC Sum			
		t_0_	t_1_	t_2_	D	t_0_	t_1_	t_2_	D	t_0_	t_1_	t_2_	D	t_0_	t_1_	t_2_	D
1	CR	3.4	5.3	4.6	↔	0.8	3.2	6.8	↔	3.3	4.1	2.9	↔	0.37	0.34	0.36	↔
2	CR	3.6	2.3	5.5	↔	5.2	4.2	8.9	↔	4.9	5.5	4.3	↔	0.48	0.57	0.24	↔
3	PR/ PD	5.7	2.9	**10.3**	↑	**29.1**	17.7	**38.7**	↑	**1.9**	6.7	2.5	↔	0.27	0.17	**0.08**	↓
4	PR/ PD	3.4	3.1	**15.3**	↑	4.4	2.1	7.9	↔	2.7	3.7	**0.7**	↓	0.31	0.31	**0.01**	↓
5	SD	3.4	5.4	3.0	↔	17.5	12	9.1	↔	3.7	2.6	7.5	↔	0.15	0.28	0.32	↔
6	CR	**7.2**	2.6	3.5	↔	6.9	1.1	1.3	↔	10	13.5	8.7	↔	**0.05**	0.17	0.16	↔
7	SD	5.0	3.3	2.6	↔	4.4	5.6	0.9	↔	3.2	4.6	5.8	↔	0.175	0.39	0.24	↔
8	PR	2.0	2.9	1.2	↔	8.2	7	3.4	↔	4.9	2.7	5.5	↔	0.36	0.46	0.46	↔
9	PR	2.8	4.4	2.6	↔	11	5.7	13.1	(↑)	10.5	7.3	8.4	↔	0.24	0.31	0.22	↔
10	PR	3.9	3.2	4.5	↔	9.7	6.4	8.2	↔	4.8	5.4	5.1	↔	0.22	0.27	0.20	↔
11	PR	**8.7**	4.4	2.7	↔	**22.3**	11	6.9	↔	**0.3**	2.2	3.5	↔	**0.03**	0.13	0.19	↔
12	CR	1.9	2.3	3.3	↔	6.3	7.4	1.7	↔	11.8	11.8	11.8	↔	0.22	0.15	0.16	↔

**Table 3:** Alterations in the ratio of slan+/HLA-DR^low^ monocytes over time. The quotients of slan+ non-classical monocytes and of HLA-DR^low^ MDSC (both as % of monocytes) were determined in the 12 patients at baseline (t_0_), the time point of third ICI application (t_1_), and after at least 12 months of OS (t_2_). Patients still alive at the end of the study are highlighted in yellow. Patients with a very low quotient, e.g., a preponderance of HLA-DR^low^ MDSC, are marked in grey.

Patient No.	Slan+/HLA-DR^low^ Mono t_0_	slan+/HLA-DR^low^ Mono t_1_	slan+/HLA-DR^low^ Mono t_2_
**1**	4.13	1.29	0.43
**2**	0.94	1.31	0.48
**3**	0.07	0.38	0.06
**4**	0.61	1.76	0.09
**5**	0.21	0.22	0.82
**6**	1.45	12.27	6.69
**7**	0.73	0.82	6.44
**8**	0.60	0.39	1.62
**9**	0.95	1.28	0.64
**10**	0.72	0.63	0.76
**11**	0.01	0.20	0.51
**12**	1.87	1.59	6.94

## Data Availability

All the data of this study are available upon request.
